# Influence of Controlled Lighting Conditions on Smartphone-Based Augmented Reality Recognition of a 3D-Printed Dental Model for Implantological Applications

**DOI:** 10.3390/jfb17080384

**Published:** 2026-08-04

**Authors:** Michael Moncher, Christoph Paul, Adrian Schletter, Lojine Elbialy, Constantin von See

**Affiliations:** Research Center for Digital Technologies in Dentistry and CAD/CAM, Department of Dentistry, Faculty of Medicine and Dentistry, Danube Private University, Steiner Landstraße 124, 3500 Krems an der Donau, Austria; paul.christoph@dp-uni.eu (C.P.); adrian.schletter@dp-uni.ac.at (A.S.);

**Keywords:** augmented reality, dental implantology, smartphone, object recognition, computer vision, 3D-printed dental model, lighting conditions, Vuforia, contrast analysis

## Abstract

Smartphone-based augmented reality (AR) may help dentists visualize an implant axis during treatment or training. Before a virtual overlay can be displayed, however, the application must first recognize the real target reliably. This in vitro study tested how five lighting conditions affected recognition of a two-colored, 3D-printed polylactic acid (PLA) dental model by a Unity/Vuforia smartphone application. The application ran on a Samsung Galaxy S22. A GoPro HERO10 recorded the smartphone display at 240 frames per second, and the videos were evaluated frame by frame. Red, green, blue, daylight-like light-emitting diode (LED) and halogen illumination were tested at a central and an eccentric model position, with ten repetitions per condition and position. The model was not recognized under red or blue illumination within the predefined observation interval. Recognition was successful in all repetitions under green, daylight-like and halogen illumination. Recognition speed differed among these successful conditions, and the eccentric position delayed recognition under daylight-like and halogen illumination. A supplementary region-of-interest analysis also showed lighting-dependent differences in image brightness and contrast. These findings indicate that lighting should be standardized and documented when smartphone-based dental AR systems are developed and tested.

## 1. Introduction

Augmented reality (AR) combines real and virtual information in a spatially registered display and is increasingly described in medicine as a technology for visualization, navigation, training and decision support [1]. In dentistry, this concept enters a field that is already shaped by digital imaging, computer-assisted planning, additive manufacturing, dynamic navigation and data-driven methods [2,3,4]. In implantology, one central objective is to connect digital planning information with the real clinical or preclinical situation as reliably as possible.

Systematic reviews indicate that AR-assisted navigation for dental implant placement is a growing research area and may make planning information visible during treatment or training [3,5]. Mosch et al. demonstrated the feasibility of using smartphones as navigation tools for dental implant surgery [6]. Related smartphone-based applications have been developed to display implant-planning information and to support preclinical implant-placement training [7,8]. In practice, such an application could display the planned drilling axis during implant placement and provide an easy-to-use aid in complex or esthetically demanding cases. The same visual guidance can also help students learn how to plan and assess an implant axis.

A virtual overlay is useful only if the application first recognizes the real model or anatomical region. If recognition is delayed, unstable or absent, the planned implant axis or virtual tooth cannot be displayed reliably. Object recognition is therefore a necessary first step in a smartphone-based dental AR workflow.

Three-dimensionally printed dental models provide a reproducible and standardized environment for such preclinical investigations. Additive manufacturing is established in dentistry and enables models in which geometry, surface characteristics, color and clinically relevant target regions can be controlled [9,10]. From the perspective of functional biomaterials, such a model is not only a passive test object but also part of a digital workflow in which material surface, model color, camera system and software-based recognition interact.

Lighting is an important factor in camera-based recognition. The recorded color and brightness depend on the object, the spectrum and intensity of the light source, and the spatial distribution of the illumination [11]. Color information can help identify an object, but its value changes when the illumination changes [12]. Brightness, contrast, noise and low-light conditions can also affect object detection and tracking [13,14,15]. In dental smartphone photography, both the acquisition conditions and the device can influence recorded color values [16,17]. Directly comparable dental studies are scarce. However, a mobile Unity/Vuforia application for equipment training was tested at different illuminance levels and showed that focus and device-related factors affected detection [18]. Studies of fiducial-marker detection have likewise reported reduced reliability under difficult or non-uniform lighting [19,20].

Against this background, the present study examined how defined lighting conditions influenced recognition of a 3D-printed dental model by the Unity/Vuforia application used in an implantological AR workflow. Vuforia was selected because it formed the recognition component of the existing smartphone application, not because it was intended to represent every possible AR algorithm. Red, green and blue illumination represented distinct spectral conditions available from the LED system, whereas daylight-like and halogen illumination represented broader light conditions closer to common working illumination. The study assessed recognition rate and frames until recognition at two model positions. A supplementary region-of-interest (ROI) analysis was used to describe brightness and contrast within the relevant model area.

## 2. Materials and Methods

### 2.1. Study Design

This experimental in vitro study examined whether lighting affects recognition of a 3D-printed dental model by a smartphone-based AR application. The setup was designed so that illumination, model position and video recording could be controlled and repeated. The study evaluated the recognition step that must occur before the application can display a virtual tooth and a planned implant axis.

**Figure 1:**
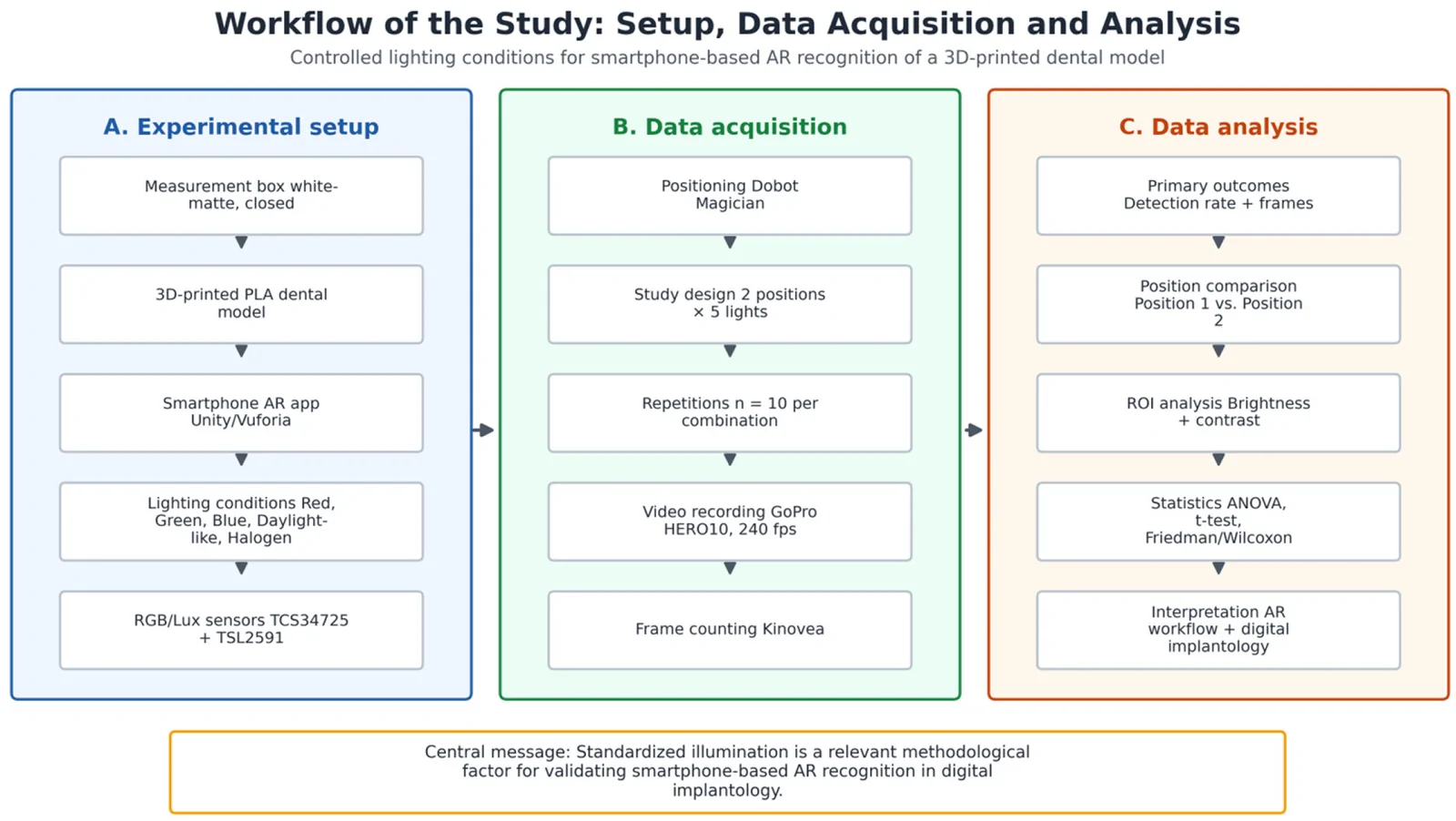
Workflow of the study. The experimental procedure was divided into setup preparation, data acquisition and data analysis. The workflow included the controlled measurement box, the 3D-printed PLA dental model, the smartphone-based Unity/Vuforia AR application, five lighting con ditions, standardized positioning, high-speed video recording, frame counting, ROI-based image analysis and statistical evaluation.

Figure 1 summarizes the study workflow. For clarity, the main components are described here by function: a smartphone-based AR application developed in Unity with Vuforia, a robotic positioning system, an external high-speed camera, RGB and illuminance sensors, and video-analysis software for frame-by-frame evaluation. The specific devices, software versions and functions are described in Section 2.3, Section 2.4, Section 2.5, Section 2.6, Section 2.7, Section 2.8, Section 2.9, Section 2.10 and Section 2.11. The workflow comprised setup preparation, data acquisition and data analysis. The model was recorded in two standardized positions under five lighting conditions, with ten repetitions per condition and position.

The primary outcome was the number of recorded frames from illumination onset until visible recognition. Because all videos were recorded at the same high frame rate, fewer frames indicated faster recognition. If recognition did not occur within the predefined observation interval, 1000 frames were recorded as a timeout or non-recognition value.

Measurements were performed for five lighting conditions and two defined object positions. Ten repeated measurements were obtained for each combination of lighting condition and position. Repetition was used to capture random variation in the recognition process and to allow statistical comparison between measurement conditions.

### 2.2. Test Object

The test object was a two-colored, 3D-printed dental model with a tooth gap and a potential implant region. The teeth were white, and the gingival part was red, creating visible color and structural transitions that could be used by the camera-based recognition system.

The model was manufactured from PLA by fused deposition modeling. It was used directly after printing, without coating or surface treatment, and was dry and visually matte during the experiment. The model provided a standardized object with constant geometry and reproducible positioning. It was intended as a controlled preliminary test object and not as a complete simulation of teeth, gingiva or the moist oral environment. Further printing parameters, such as layer height or exact printer settings, were not available for this study.

The model served as a Vuforia Model Target for the smartphone application. The application was designed to recognize the target region and display a virtual tooth and planned drilling axis for implantological orientation. The intended long-term use is simple visual support during implant placement, especially in complex or esthetically demanding cases, and demonstration or preclinical training for students. The present study evaluated only the preceding recognition step and did not evaluate surgical placement accuracy.

### 2.3. Smartphone, Application and Recognition Principle

Recognition was performed using a Samsung Galaxy S22 smartphone (Samsung Electronics Co., Ltd., Suwon, Republic of Korea) running Android 14 and One UI 6.1 at the time of documentation [21]. The Samsung Galaxy S22 was selected because the pre-existing Android application was already implemented and operational on this device and because it fulfilled the hardware and software requirements of the Unity/Vuforia workflow. Only one smartphone model was used to keep the camera hardware, automatic image processing and operating-system conditions constant; consequently, device-to-device generalizability was not assessed. The custom application was developed with Vuforia Engine (PTC Inc., Boston, MA, USA) [22,23,24] and implemented in Unity Editor 2022.3.9f1 (Unity Technologies, San Francisco, CA, USA) [25,26]. Unity and Vuforia were selected because the pre-existing smartphone-based implantological AR application had been developed using this platform combination: Unity provided the application and visualization framework, whereas Vuforia enabled Model Target-based recognition and tracking of the predefined three-dimensional dental model. Accordingly, the study evaluated this specific AR workflow under controlled lighting conditions and was not intended to compare different AR platforms or recognition algorithms. The Vuforia Engine version could not be determined retrospectively.

The dental model was prepared as a Vuforia Model Target. This means that the application compared the live camera image with previously generated reference information from the known model [22,23,24]. The system therefore did not attempt to identify an unknown object. It searched for the prepared model and then tracked its position in the camera image.

No visible QR code or separate fiducial marker was attached to the model. Recognition instead depended on model-related information such as shape, edges, surface structure and brightness or color transitions. Once the model was recognized, the virtual overlay was linked to this known target rather than placed freely in space.

Successful recognition was visible on the smartphone display as the projection of a virtual tooth and the proposed implant axis into the tooth gap. This visible event was used as the recognition endpoint. The endpoint was evaluated in the external video rather than from an internal application timestamp, allowing retrospective frame-by-frame assessment.

The application remained open throughout the complete measurement series and was not restarted between individual measurements or lighting blocks. The closed setup was monitored wirelessly and by the external video recording.

Autofocus, exposure, ISO and white balance could not be manually fixed within the application. The camera was not tapped or otherwise adjusted during measurement, and all camera settings remained under automatic control. This reflects the intended simple clinical use of the application, but it also means that automatic exposure, focus, white-balance and image-processing responses may have differed between lighting conditions. The experiment therefore measured the performance of the complete smartphone–camera–application system rather than the isolated effect of the light source on the recognition algorithm.

### 2.4. Measurement Environment

Measurements were performed in a custom-built closed measurement box. The box measured 40 cm × 40 cm × 40 cm. The interior was painted matte white. The closed box shielded the experimental setup from variable ambient light and was intended to reduce external confounding factors.

The white matte inner surface was chosen to support diffuse reflection and a more homogeneous light distribution inside the box. In optical metrology, integrating spheres are used as reference systems because their diffusely reflecting interior enables a more spatially uniform radiation or light distribution and supports photometric measurements and calibration [27,28]. Although the measurement box used in the present study was not an integrating sphere in the strict metrological sense, the underlying principle of a bright, matte and diffusely reflecting surface was used to reduce directed reflections, harsh highlights and the influence of variable ambient illumination.

The closed measurement environment was essential for the research question because the object recognition of a camera-based system depends strongly on the information available in the image. Changes in illumination, brightness, light color or shadow formation can alter the appearance of edges, surface structures and color transitions. No relevant visible shadow formation was observed in the model region during the measurements. Nevertheless, subtle differences in light distribution, surface brightness or camera-internal exposure cannot be fully excluded.

**Figure 2:**
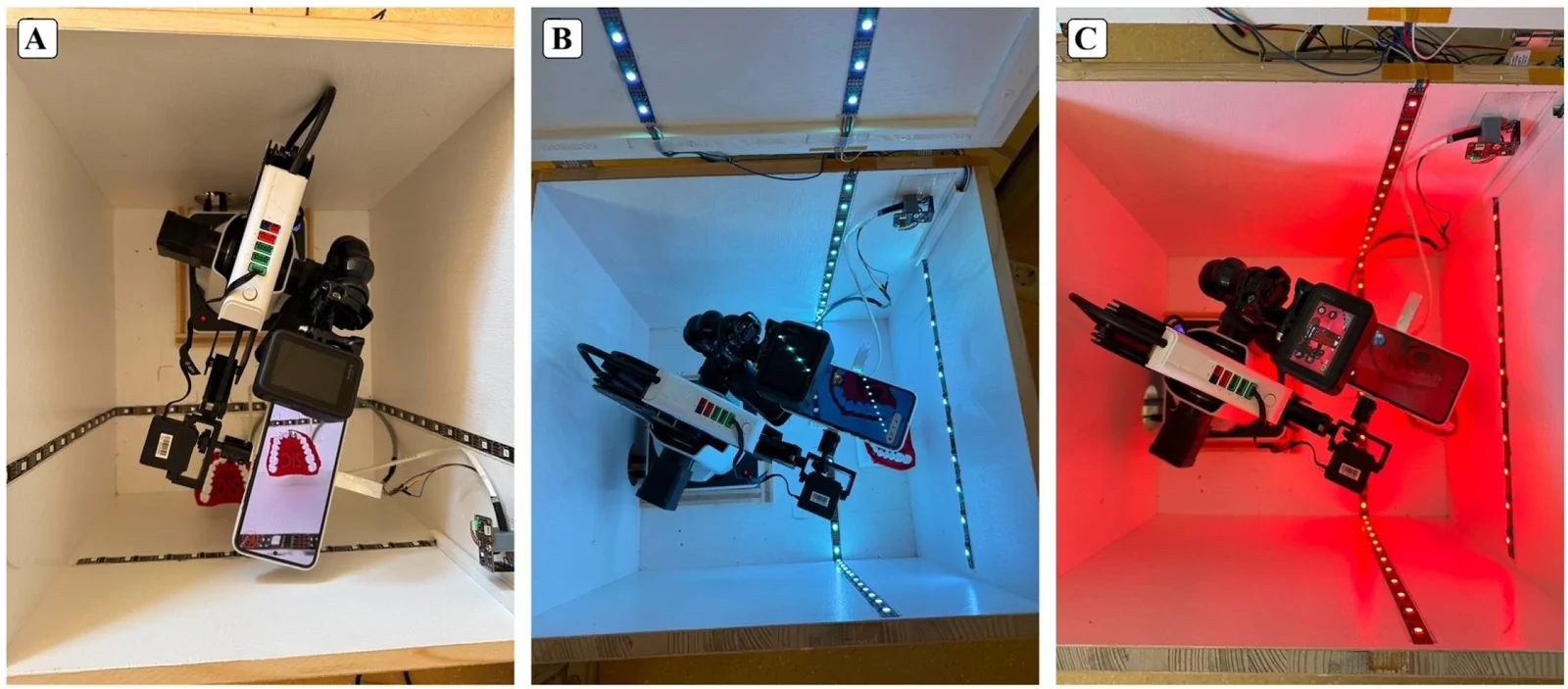
Representative photographs of the experimental setup inside the closed measurement box. (**A**) Setup without active colored illumination. (**B**) Setup under daylight-like LED illumination. (**C**) Setup under red LED illumination. The images show the matte-white measurement box, the mounted LED strips, the Dobot-based positioning system, the Samsung Galaxy S22 running the AR application, the GoPro HERO10 used for external recording of the smartphone display and the 3D-printed dental model.

Representative photographs of the experimental setup are shown in Figure 2. The images illustrate the closed matte-white measurement box, the mounted LED strips, the Dobot-based positioning system, the smartphone running the AR application, the GoPro HERO10 [29] used for external recording of the smartphone display and the 3D-printed dental model. The setup is shown without active colored illumination and under exemplary daylight-like and red LED illumination. These photographs document the spatial arrangement of the main components and the controlled lighting environment used during data acquisition.

### 2.5. Lighting System and Sensor Characterization

Defined illumination was generated with a WS2801-based addressable RGB LED strip system (Shenzhen BTF-Lighting Technology Co., Ltd., Shenzhen, China) [30]. Red, green and blue were selected as distinct spectral conditions that could be generated reproducibly with the LED system. Daylight-like LED and halogen illumination were included as broader light conditions closer to common working illumination. These five conditions were used to test how the dental model and the complete smartphone recognition system behaved under clearly different lighting situations; the narrow-band conditions were not intended as recommended clinical illumination.

The daylight-like condition was produced by mixing the available LED channels. It was an artificial approximation and not natural daylight; therefore, the term daylight-like illumination is used throughout the manuscript. Color-temperature values were interpreted only when they were technically plausible.

For halogen illumination, an OSRAM HALOPIN OVEN G9 halogen lamp (25 W, 230 V, 2700 K; GTIN 4008321703552; LEDVANCE GmbH, Garching near Munich, Germany) with a 35° beam angle was used. This additional condition was included to compare the LED-based conditions with a conventional light source of different spectral characteristics. Possible interactions between mains-powered light sources, image acquisition and mains frequency were not investigated as a primary methodological endpoint but may be relevant when interpreting the results.

Two sensors documented the lighting conditions. A TCS34725 RGB color sensor with an IR filter (Adafruit Industries, New York, NY, USA) recorded red, green, blue and clear-light values [31,32]. A TSL2591 digital light sensor, Product ID 1980 (Adafruit Industries, New York, NY, USA), measured illuminance in lux [33,34].

Using both sensors provided a numerical description of each lighting condition instead of relying only on labels such as red, green, blue, daylight-like or halogen. The sensor readings also allowed the dark starting condition to be checked before each measurement.

The sensor values were used before or during the experimental series to control the LED settings and document illumination. For analysis, the RGB and lux values recorded during the active illumination phase were considered. The CCT column contained in the log files was not used as a secure primary parameter, because color-temperature values under monochromatic illumination and in individual halogen logs were only partly plausible or not stably interpretable.

### 2.6. Control and Data Acquisition

A Raspberry Pi 4 Model B single-board computer (Raspberry Pi Ltd., Cambridge, UK) [35] was used to control the LED illumination, read the sensors and technically supervise the measurement environment. Python was used for programming. The control and measurement scripts were executed on the Raspberry Pi in a Python 3.11.2 environment [36].

I2C communication was organized using a PCA9548 8-channel STEMMA QT/Qwiic I2C multiplexer, TCA9548A-compatible, Product ID 5626 (Adafruit Industries, New York, NY, USA) [37]. Hardware communication was implemented using Adafruit CircuitPython libraries according to the Adafruit installation workflow for Raspberry Pi/Blinka [38]. The documented versions were adafruit-circuitpython-ws2801 0.10.18 for LED control [39], adafruit-circuitpython-tcs34725 3.3.20 for the color sensor [32], adafruit-circuitpython-tsl2591 1.4.2 for the light sensor [34] and adafruit-circuitpython-tca9548a 0.8.0 for the I2C multiplexer [40]. The use of established libraries was intended to support standardized communication with the components and improve reproducibility of the measurement process.

The Raspberry Pi and LED components were powered by an LRS-100-5 switching power supply, 5 V DC, 18 A, 90 W (MEAN WELL Enterprises Co., Ltd., New Taipei City, Taiwan) [41]. Stable DC power supply was relevant because voltage fluctuations can influence LED brightness and color stability. In a digitally controlled RGB or RGBW LED system, stable power contributes to the reproducibility of the selected illumination conditions.

### 2.7. Positioning and Definition of Measurement Positions

Reproducible positioning was achieved using a Dobot Magician robotic arm (Shenzhen Yuejiang Technology Co., Ltd., Shenzhen, China) [42]. The positions of the robot and the attached recording unit could be documented with spatial X, Y and Z coordinates in DobotStudio. This allows the measurement positions to be reproduced for additional measurements or future experimental setups.

Two measurement positions were defined. Position 1 represented the target object centrally in the image field of the smartphone application and corresponded to an idealized recording situation. Position 2 deliberately placed the object outside the image center. This eccentric position was selected to examine a less ideal but more application-oriented situation. In practical smartphone-based use, a relevant anatomical structure or target region is not necessarily located exactly in the center of the camera frame.

By comparing both positions, it was possible to assess whether recognition performance was influenced only by lighting condition or also by the position of the target object within the camera frame. This is particularly relevant for reference- or track-point-based AR applications because the features used by the system may be visible to different degrees depending on image section, perspective and object location.

### 2.8. Video Recording and Measurement Procedure

Recognition on the smartphone was not documented by internal screen recording but by filming the smartphone display with a separate camera. A GoPro HERO10 Black action camera (GoPro, Inc., San Mateo, CA, USA) [29] was used for this purpose. The camera was operated at 240 frames per second with a wide-angle setting. Based on the available information, the resolution was probably 2.7 K; because this setting was not additionally documented, it is reported only as the probable device setting.

The GoPro was attached to the robot system to maintain a constant camera position relative to the smartphone display. The alignment was selected so that the smartphone display was fully and clearly visible during the measurements. This was essential because successful object recognition was determined by the visible projection of a virtual tooth and implant axis on the smartphone display.

The high frame rate was selected to determine the recognition event with high temporal resolution. Wireless control and transmission of the camera also enabled monitoring of the procedure inside the closed measurement box without opening the measurement environment during data acquisition.

Measurements were performed in the same fixed order at both model positions: red, green, blue, daylight-like and halogen. All ten repetitions for one lighting condition were completed before the next condition was prepared. This block design reduced repeated reconfiguration of the illumination system and therefore reduced handling effort and potential setting errors. The order was not randomized, because every individual measurement was restarted from a controlled dark baseline rather than from the preceding illuminated state.

Before each repetition, the illumination was switched off completely until the camera image was dark and the sensors confirmed that the baseline condition had been restored. The selected illumination was then switched on again. No additional waiting period was used. The last frame with the light still off was defined as the start frame, and frames were counted until the visible recognition event occurred.

### 2.9. Manual Frame-Based Evaluation

The recognition videos were evaluated manually using the freely available video-analysis software Kinovea, version 2025.2, obtained via the Microsoft Store [43]. Kinovea allows frame-by-frame inspection of video files and is therefore suitable for determining defined events within a recording.

For each measurement, the start point was first defined. The start frame was the last frame in which the respective illumination was still switched off. The video was then inspected frame by frame until the smartphone application displayed successful recognition by projecting the virtual tooth and implant axis into the tooth gap. The difference between start frame and recognition frame yielded the number of frames until object recognition. If no recognition became visible within the observation interval, the threshold value of 1000 frames was documented.

### 2.10. ROI-Based Contrast Analysis

In addition to recognition performance, an ROI-based image analysis was performed. ROI stands for region of interest and refers to a selected image area used for evaluation. In this study, a polygonal region containing relevant parts of the dental model was manually defined for each of the two measurement positions. Restricting the analysis to an ROI was intended to prevent irrelevant image areas, such as borders or background regions, from disproportionately influencing the analysis of image quality.

**Figure 3:**
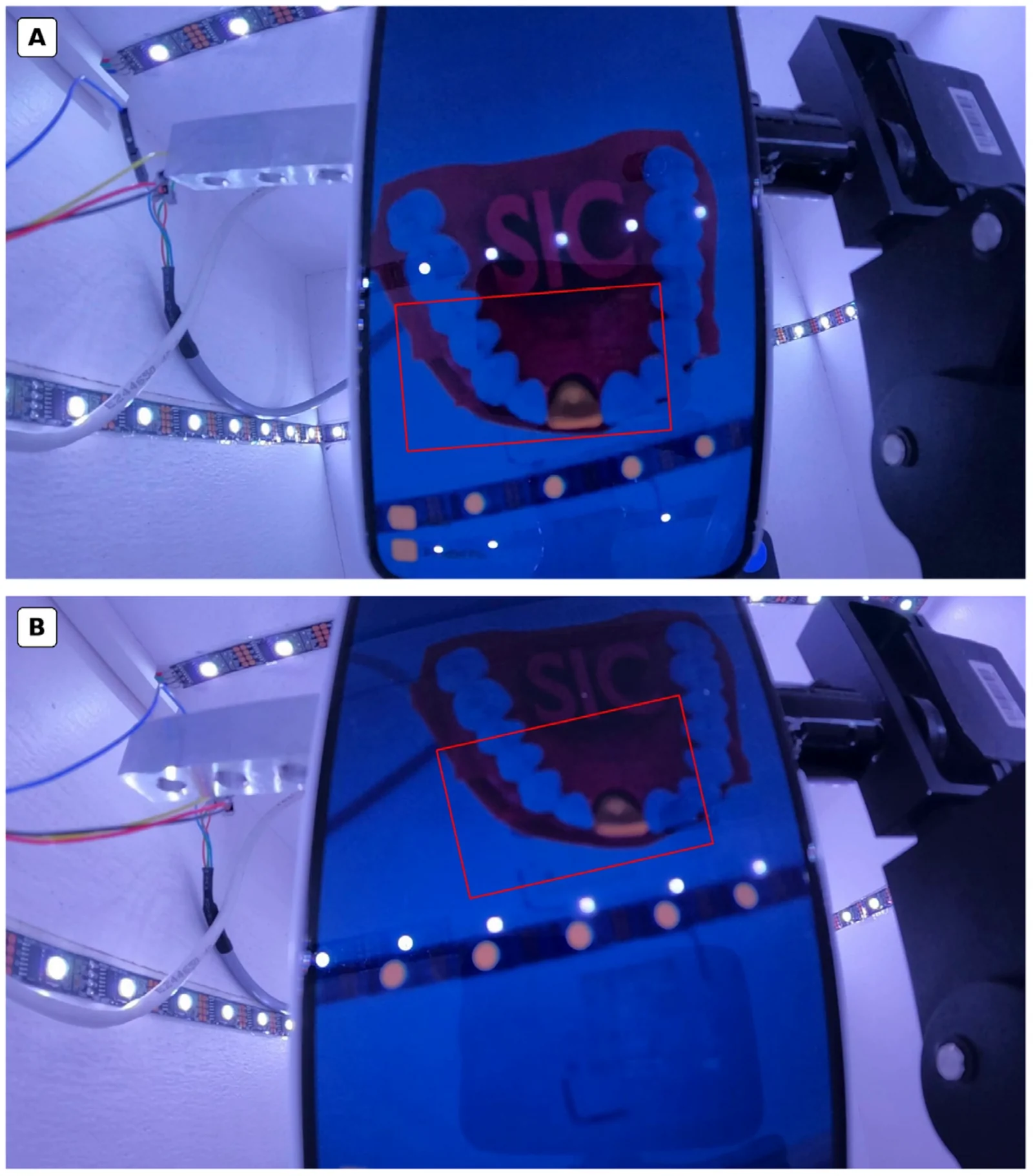
Definition of the regions of interest for the supplementary image analysis. (**A**) ROI used for position 1. (**B**) ROI used for position 2. The red polygonal outlines mark the image regions from which brightness, standard deviation, contrast and robust Michelson contrast values were calculated.

The manually defined ROIs used for the supplementary contrast analysis are shown in Figure 3. The red polygonal outlines indicate the image regions evaluated for position 1 and position 2. These regions included the relevant parts of the dental model and were selected to exclude irrelevant background areas, borders and peripheral image regions from the brightness and contrast analysis.

The ROI-based contrast analysis was performed using a custom Python script. Individual frames were read with OpenCV and converted to grayscale. A binary mask was then created from the manually defined polygon for the respective position so that only pixels within the ROI were evaluated. ROI coordinates were referenced to a resolution of 2048 × 1151 pixels and were proportionally scaled for images of different size.

Several parameters were calculated within the ROI. Mean brightness described the average grayscale value of the pixels within the ROI. The standard deviation of pixel values described the spread of brightness values and may indicate brightness differences, edges, textures or structural image information. In addition, contrast was calculated as the difference between the maximum and minimum pixel value.

Classical Michelson contrast was calculated from the maximum and minimum intensity value within the ROI. Because this value can be sensitive to single extremely bright or dark pixels, a robust contrast parameter was also calculated. The 1st and 99th percentiles of pixel intensities were used for this purpose. Robust contrast was calculated as the difference between p99 and p1, and robust Michelson contrast as the quotient of this difference and the sum of p99 and p1. This reduced the influence of outliers at the upper and lower ends of the brightness distribution.

The ROI-based analysis was not used as the primary endpoint of the investigation but as a supplementary image-analytic description of image quality. It was intended to assess whether lighting conditions with better or poorer recognition also showed measurable differences in brightness and contrast within the relevant image region.

### 2.11. Software for ROI and Statistical Analysis

ROI evaluation was performed in Python 3.14.4 (Python Software Foundation, Wilmington, DE, USA) [44]. Image processing used OpenCV via the opencv-python package, version 4.13.0.92 [45,46]. Numerical calculation of pixel values, percentiles and contrast parameters was performed with NumPy 2.4.4 [47]. Results were exported as Excel files using OpenPyXL 3.1.5 [48]. Modules from the Python standard library, including pathlib for file-path handling and re for pattern-based file sorting, were also used.

The statistical analysis was descriptive and inferential. For each combination of lighting condition and position, measurements were first summarized descriptively. Mean, standard deviation, median, minimum and maximum were calculated to describe the distribution of frame counts and contrast parameters.

To assess whether lighting condition significantly influenced recognition performance, one-way analysis of variance was performed for the primary outcome, the number of frames until successful recognition. Analysis of variance (ANOVA) was used because more than two lighting conditions were compared. Paired *t*-tests were used for pairwise comparisons between the two positions within the same lighting condition.

For the supplementary ROI parameters, the data distribution was examined first. Because these image parameters were not consistently normally distributed and were obtained from consecutive video frames, they were reported mainly descriptively. Exploratory comparisons used a Friedman test followed by pairwise Wilcoxon tests with Holm correction.

The significance level was set at alpha = 0.05. Values of *p* < 0.05 were interpreted as statistically significant. Analyses were performed with SigmaPlot, version 13 (Systat Software Inc., San Jose, CA, USA) [49], and Microsoft Excel for the web via a Microsoft 365 university account (Microsoft Corporation, Redmond, WA, USA) [50]. The ROI contrast data were additionally preprocessed in Python and then merged in tabular form. Because consecutive video frames may not be fully independent, the ROI statistics were interpreted as supplementary image-analytic descriptions rather than as a primary proof of efficacy.

Special methodological attention was given to values of 1000 frames. This value represented the predefined threshold for absent recognition and was interpreted as timeout or non-recognition. For lighting conditions in which all repetitions reached 1000 frames, no within-group variability was present. Statistical tests requiring group variance are therefore only partly meaningful for these groups. These findings were therefore considered separately in the discussion.

## 3. Results

### 3.1. Data Basis and Evaluation Logic

Ten repeated measurements were evaluated for each combination of lighting condition and measurement position. Five lighting conditions were investigated: red, green, blue, daylight-like illumination and halogen illumination. Measurements were performed at two defined positions. Position 1 represented a central presentation of the 3D-printed dental model in the camera frame, whereas position 2 represented an eccentric presentation outside the image center.

The primary outcome parameter was the number of frames until visible object recognition by the smartphone-based AR application. Successful recognition was clearly indicated by projection of a virtual tooth and the proposed implant axis into the tooth gap of the model. Under the constant recording conditions, a lower frame count corresponds to faster recognition. The value of 1000 frames represented the predefined threshold and was interpreted as timeout or non-recognition.

Because the recording was performed at 240 frames per second, one frame corresponded to approximately 4.17 ms. The threshold of 1000 frames therefore corresponded to approximately 4.17 s. Frame counts were retained as the primary measurement unit; conversion into seconds served only for temporal interpretation.

In addition to recognition performance, ROI-based image analysis was performed as a supplementary characterization of image quality. The main parameters of interest were robust Michelson contrast, mean brightness and standard deviation of pixel values within the defined region of interest.

### 3.2. Characterization of Lighting Conditions

Table 1 summarizes the measured RGB values and illuminance. Daylight-like illumination showed the highest illuminance (900.8 +/− 2.4 lux); the other conditions ranged from 325.4 to 549.3 lux. CCT was interpreted only for the daylight-like condition (approximately 5509 +/− 166 K) because CCT values under monochromatic and halogen illumination were not sufficiently reliable.

The recognition pattern did not parallel illuminance alone. Although daylight-like illumination had the highest measured illuminance, reliable recognition was also obtained under green and halogen illumination at lower illuminance levels. Conversely, red and blue illumination did not produce recognition despite measurable illuminance. This descriptive comparison separates the recorded light intensity from the categorical recognition outcome.

### 3.3. Recognition Rate by Lighting Condition

Recognition showed a complete separation between conditions (Table 2). Red and blue illumination produced no recognition at either position, whereas green, daylight-like and halogen illumination produced recognition in all repetitions.

When the two positions were considered together, this corresponded to 0 of 20 successful trials for red and blue illumination and 20 of 20 successful trials for green, daylight-like and halogen illumination. Thus, the principal outcome was not a small shift in recognition frequency but a complete separation between unsuccessful and consistently successful lighting conditions within the predefined observation interval.

### 3.4. Frame Count Until Recognition at the Central Position

At the central position, halogen illumination produced the lowest mean frame count, followed by daylight-like and green illumination (Table 3). Red and blue illumination produced only timeout values.

The overall ANOVA was significant and was strongly influenced by the timeout conditions. When only the three successfully recognized conditions were analyzed, the difference remained significant (*p* = 0.018). Halogen differed significantly from green; the other pairwise comparisons were not significant.

For the three successful conditions, the mean +/− SD frame counts were 130.6 +/− 22.5 under halogen, 162.0 +/− 37.2 under daylight-like and 173.6 +/− 36.0 under green illumination. Daylight-like illumination therefore occupied an intermediate descriptive position. The pairwise results supported a difference between halogen and green, whereas neither successful condition differed significantly from daylight-like illumination.

### 3.5. Frame Count Until Recognition at the Eccentric Position

At the eccentric position, green illumination produced the lowest mean frame count, followed by halogen and daylight-like illumination (Table 4). Red and blue again produced only timeout values.

The difference among the three successfully recognized conditions remained significant (*p* < 0.001). Green produced significantly lower frame counts than both halogen and daylight-like illumination, whereas halogen and daylight-like illumination did not differ significantly.

At this position, the mean +/− SD frame counts were 153.5 +/− 27.8 under green, 181.8 +/− 16.9 under halogen and 196.8 +/− 12.0 under daylight-like illumination. In contrast to the central position, green was therefore the fastest successful condition. The inferential comparisons supported this descriptive ordering for green versus both other successful conditions.

### 3.6. Comparison Between Central and Eccentric Model Position

The effect of object position depended on the lighting condition (Table 5). Under green illumination, the difference between positions was not significant (*p* = 0.225). The eccentric position required significantly more frames under daylight-like (*p* = 0.022) and halogen illumination (*p* = 0.001). No position test was meaningful for red or blue because recognition failed at both positions.

The mean change from position 1 to position 2 was −20.1 frames under green illumination, +34.8 frames under daylight-like illumination and +51.2 frames under halogen illumination. Accordingly, eccentric presentation was not associated with a uniform delay across lighting conditions: the direction was slightly favorable under green but unfavorable under daylight-like and halogen illumination. For red and blue illumination, the constant timeout values describe persistent non-recognition rather than equivalent recognition speed.

### 3.7. ROI-Based Contrast Analysis

Robust Michelson contrast differed clearly among lighting conditions (Table 6). At position 1, the ranking was green > daylight-like > halogen > red > blue. The Friedman test was significant (chi-square = 3881.016; df = 4; *p* < 0.001), and all pairwise Wilcoxon comparisons remained significant after Holm correction (each *p* < 0.001).

The mean robust Michelson contrast values at position 1 were 0.8264 +/− 0.0268 for green, 0.8123 +/− 0.0089 for daylight-like, 0.6584 +/− 0.0104 for halogen, 0.4796 +/− 0.0499 for red and 0.3753 +/− 0.0263 for blue illumination. None of the five distributions was normally distributed; therefore, the nonparametric global and pairwise comparisons were retained. The numerical ordering was consistent for the mean and median values reported in Table 6.

### 3.8. Descriptive ROI Findings Across Both Positions

Across both positions, blue illumination showed the lowest brightness and contrast dispersion. Green and daylight-like illumination showed high robust contrast, whereas halogen values varied more with model position. These findings are descriptive and exploratory because consecutive video frames are temporally related and cannot be treated as fully independent observations.

The cross-position values provide additional detail not contained in Table 6. Under blue illumination, mean brightness was 12.84 grayscale levels at position 1 and 17.02 at position 2, while the corresponding standard deviations were 2.73 and 4.44. Robust Michelson contrast decreased from approximately 0.826 at position 1 to 0.673 at position 2 under green illumination but increased from approximately 0.658 to 0.742 under halogen illumination. These opposite position-related changes reinforce the descriptive character of the ROI analysis and show that the effect of object position differed among lighting conditions.

### 3.9. Summary of Main Results

In summary, recognition failed under red and blue illumination but was reliable under green, daylight-like and halogen illumination. The fastest successful condition depended on model position, and the ROI analysis confirmed lighting-dependent differences in image contrast. Detailed recognition results are provided in Table 2, Table 3, Table 4 and Table 5; Table 6 reports robust Michelson contrast at position 1, while selected cross-position ROI values are reported in Section 3.8.

## 4. Discussion

### 4.1. Summary of Central Findings

This in vitro study examined a necessary first step in a smartphone-based implantological AR workflow: the dental model must be recognized before the application can display a virtual tooth or planned drilling axis. The results describe the performance of the tested complete system, consisting of the model, lighting, Samsung Galaxy S22 camera, automatic camera processing and Unity/Vuforia application.

The main finding was a clear separation between lighting conditions with successful and unsuccessful object recognition. Under red and blue illumination, no successful recognition occurred in either measurement position within the defined observation interval. In both conditions, the maximum value of 1000 frames was reached and interpreted as timeout or non-recognition. Under green, daylight-like and halogen illumination, the model was recognized in all repeated measurements.

Object position also affected recognition, but the effect varied with lighting. The eccentric position delayed recognition under daylight-like and halogen illumination, whereas no significant positional difference occurred under green illumination. Together with the ROI analysis, these findings suggest that recognition depended not only on illuminance but on whether the camera image contained stable and usable features.

### 4.2. Recognition Approach and Relevance of Lighting

The application used a prepared Vuforia Model Target rather than free recognition of an unknown object [22,23]. Vuforia was studied because it was the recognition platform used in the existing implantological smartphone application. The aim was therefore to test this specific dental workflow under controlled lighting, not to claim that Vuforia represents all AR or machine-vision algorithms. The internal feature weighting and the relative use of color, grayscale, edge or shape information by Vuforia were not available to the authors.

Model-target recognition requires visible and repeatable information in the camera image, such as shape, edges, surface structure and brightness transitions. If lighting reduces or changes these features, recognition may be delayed or may fail. A mobile Unity/Vuforia study in another training setting also found that illumination, focus and device-related factors influenced detection [18]. Studies of fiducial markers similarly show that difficult or non-uniform lighting can reduce detection reliability, although their targets and algorithms differ from the present Model Target approach [19,20].

The two-colored PLA model contained white teeth, red gingiva and a tooth gap. Lighting could therefore change the visibility of the tooth–gingiva boundary, surface structure and target region. This interpretation is consistent with general computer-vision research showing that illumination changes object appearance and recognition-relevant image information [11,13].

### 4.3. Interpretation of Lighting-Dependent Recognition Performance

Red illumination did not have the lowest measured illuminance, so the failure under red light cannot be explained by brightness alone. A possible explanation is that red light reduced the visible difference between the red gingival part and adjacent structures, weakening model features used for matching. This was a controlled spectral condition and was not intended as recommended clinical or machine-vision illumination.

The ROI analysis showed more contrast under red than under blue illumination but less than under the three successful conditions. This indicates that a single overall contrast value cannot explain recognition. The location and type of visible features may be more important than global contrast. This explanation remains hypothetical because model color, automatic exposure, white balance and the internal Vuforia algorithm were not experimentally separated.

Blue illumination produced no recognition and showed the lowest ROI brightness and robust contrast. Low signal and weak local differences may have reduced the edges and texture information available to the recognition system. Similar losses of detection or tracking reliability under difficult lighting have been reported in computer-vision studies [14,15,19,20].

Green, daylight-like and halogen illumination allowed recognition in every repetition. Green produced the highest robust contrast and the fastest mean recognition at the eccentric position. Daylight-like illumination provided broader spectral information and preserved the tooth–gingiva transition more evenly. Halogen illumination also provided broad light but showed a stronger position effect. These explanations describe plausible image-level mechanisms; they do not identify the internal decision process of Vuforia.

### 4.4. ROI-Based Contrast Analysis and Object Position

The ROI analysis was a supplementary description of image quality rather than a direct test of the recognition algorithm. Robust Michelson contrast used the 1st and 99th percentiles instead of single minimum and maximum pixels, reducing the influence of isolated bright or dark outliers.

The contrast ranking partly matched recognition performance: green and daylight-like illumination showed high contrast and reliable recognition, whereas blue showed the lowest contrast and no recognition. Red again showed why contrast alone is insufficient, because recognition failed despite higher contrast than blue. The relevant question is whether the correct model features remain visible in the correct locations.

The comparison between position 1 and position 2 showed that object location within the camera frame can additionally influence recognition performance. Under daylight-like and halogen illumination, the eccentric position was associated with significantly higher frame counts. Under green illumination, position 2 was descriptively faster, but this difference was not significant. In an eccentric position, image-edge distortions, altered viewing angles or a different distribution of edges and contrasts may occur, potentially making matching between the real model and stored reference more difficult. This is relevant for practical use because the target region in a smartphone-based AR application is not necessarily ideally centered.

### 4.5. Relevance for Digital Dentistry and Implantological AR Workflows

The dental purpose of the application is to display a planned drilling axis during implant placement and to provide the clinician with a simple visual aid that can be integrated into treatment. Such support may be especially useful in complex cases or when implant position is important for the esthetic result. The same application can demonstrate implant-axis planning and support preclinical training for students.

Before this clinical or educational function can be used, the application must recognize and link the real target to the digital plan. The present study therefore addresses a practical reliability step that precedes evaluation of implant-placement accuracy. It also shows why the interaction among model surface, color, lighting, smartphone camera and software should be considered during validation.

Previous dental studies have focused mainly on feasibility, navigation accuracy and training with smartphone-based or other AR systems [3,5,6,7,8,51,52,53]. The present study adds a controlled assessment of recognition reliability under different lighting conditions. Because directly comparable dental studies remain scarce, the findings should be viewed as an initial system-specific contribution rather than a general rule for all dental AR platforms.

### 4.6. Technical Considerations: Smartphone Color Acquisition

Smartphone cameras usually record color through a color-filter array, such as the Bayer pattern [54]. The camera then reconstructs a full-color image from the signals of red-, green- and blue-sensitive sensor elements. Narrow-band illumination can therefore provide uneven information across the color channels.

This camera principle may help explain why the image appearance changed under red, green and blue illumination. However, the present study did not examine the Galaxy S22 sensor architecture, raw RGB channels or camera-processing pipeline. It also did not reveal how Vuforia weights color channels or other image features. The Bayer explanation must therefore remain a possible technical interpretation and not a proven mechanism.

### 4.7. Strengths and Limitations

A strength of the study is the controlled experimental setup. The closed, matte-white measurement box reduced variable ambient lighting and enabled reproducible illumination situations. Lighting conditions were described not only qualitatively but also by RGB and lux values. Reproducible positioning using the Dobot Magician enabled standardized evaluation of a central and an eccentric position. The external high-speed recording at 240 fps allowed fine temporal resolution and retrospective verification of the recognition time. In addition, the visible projection of a virtual tooth and implant axis provided a clear recognition criterion, and the ROI-based contrast analysis added an objective description of image quality.

Several limitations should be considered. First, the study used one untreated, dry and matte two-colored PLA model. PLA differs from enamel, dentin, gingiva and moist oral tissues in color, translucency, gloss, light scattering and surface reflection. The model was deliberately used as a standardized preliminary test object and not as a complete simulation of the oral cavity. In a patient, saliva and wet surfaces can create specular reflections, while blood, instruments, lips, cheeks or the tongue may cover relevant features. Patient and soft-tissue movement, restricted access and variable ambient or operating light may further affect focus, visibility and target recognition.

Second, only one smartphone model and one Unity/Vuforia application were tested. Other cameras, operating systems, software versions or AR platforms may process color, exposure and target features differently. Autofocus, exposure, ISO and white balance could not be fixed in the application and remained automatic throughout the experiment. Automatic exposure may compensate for brightness, white balance may alter color differences, and autofocus or internal image enhancement may respond differently to each lighting condition. Consequently, the results cannot be attributed solely to the external light source and instead describe the behavior of the complete tested system. The internal Vuforia recognition algorithm was not accessible, so the relative contribution of color, grayscale, edges and shape could not be determined.

Third, the lighting order was block-based and identical at both positions. The sequence was red, green, blue, daylight-like and halogen. The design reduced repeated configuration steps and potential setting errors, and the light was fully switched off before every repetition until the sensors confirmed restoration of the dark baseline. Nevertheless, randomization was not performed, so residual sequence, adaptation or application-state effects cannot be completely excluded. The application was not restarted between blocks. The timeout value of 1000 frames and the temporally related ROI frames are further limitations; therefore, ROI statistics were interpreted as exploratory.

### 4.8. Outlook

Future studies should separate the effects of illumination from automatic camera adaptation by comparing automatic and manually fixed camera settings and, where possible, by examining raw RGB-channel information. Testing several smartphones, operating systems, Vuforia versions and alternative AR platforms would show whether the present findings are device- or software-specific.

More clinically realistic studies should include tooth- and gingiva-like materials with different translucency, gloss and surface texture, as well as wet surfaces, saliva substitutes, soft-tissue movement, instruments and changing dental operating lights. Randomized lighting order and independent restarting of the application could further reduce possible sequence and application-state effects.

Further work should also evaluate recognition and tracking after the initial target is found, including tracking loss, recovery time, accuracy of the displayed drilling axis and reproducibility of the virtual overlay. These steps are required before the application can be assessed as a clinical navigation aid. Table 7 summarizes the recognition outcomes and the possible image-related explanations for each lighting condition.

**Table 7:** Summary interpretation of the lighting conditions.

Lighting Condition	Recognition	Possible Image-Related Explanation
Red	No recognition	Possible weakening of relevant red–white transitions between gingiva and tooth; reduced differentiation of model-relevant features.
Blue	No recognition	Low brightness and low contrast dispersion within the ROI; presumably too few stable image features.
Green	Reliable recognition	High robust Michelson contrast and sufficient representation of relevant structures.
Daylight-like	Reliable recognition	More balanced light composition and good preservation of color and edge information.
Halogen	Reliable recognition	Broader spectral characteristics and sufficient feature visibility; position-dependent differences possible.

## 5. Conclusions

Smartphone-based AR may provide a simple way to display implant-planning information during treatment or training. However, the real target must first be recognized reliably. This study examined the initial recognition step in one controlled system consisting of a two-colored PLA model, a Samsung Galaxy S22 and a Unity/Vuforia application.

The results show that the Vuforia/Unity-based smartphone application did not recognize the model independently of lighting. Under red and blue illumination, no reliable recognition was achieved at either measurement position within the defined observation interval. Under green, daylight-like and halogen illumination, by contrast, the model was successfully recognized in all repeated measurements. This demonstrated a clear separation between lighting conditions that enabled stable recognition and those that led to complete non-recognition.

The supplementary ROI-based contrast analysis showed that lighting conditions also altered the measurable properties of the relevant image region. Under blue illumination, brightness and contrast parameters were particularly low. Under red illumination, color similarity between the red gingival component and red light may additionally have reduced the differentiability of relevant red–white transitions between gingiva and tooth. These findings suggest that not absolute illuminance alone, but especially the representation of usable visual features such as edges, color transitions, brightness gradients and surface structures, is crucial for recognition.

The findings add a practical methodological point to dental AR research: lighting, camera behavior, target material and software should be considered together when recognition reliability is validated. The results apply directly only to the tested model, smartphone and application and should not be generalized to all devices or AR algorithms.

Because the study used a dry PLA model in a closed measurement box, it does not reproduce the optical and dynamic conditions of the oral cavity. Future studies should include clinically relevant materials and wet surfaces, several smartphones and AR platforms, fixed camera parameters where possible, randomized lighting sequences and realistic dental illumination.

## Figures and Tables

**Table 1:** Characterization of the lighting conditions based on sensor logs.

Lighting Condition	*n*	Recorded RGB Values	Illuminance	Comment
Red	10	R 255, G 0, B 1	325.4 +/− 0.8 lux	LED condition; correlated color temperature (CCT) not interpreted
Green	10	R 0, G 86.2 +/− 5.2, B 6	520.9 +/− 1.5 lux	LED condition; CCT not interpreted
Blue	10	R 0, G 5, B 138	339.6 +/− 0.8 lux	LED condition; CCT not interpreted
Daylight-like	10	R 0, G 18.8 +/− 0.4, B 53.6 +/− 0.8	900.8 +/− 2.4 lux	Artificial LED mixture; CCT approximately 5509 +/− 166 K, interpreted cautiously
Halogen	10	R 172.2 +/− 65.9, G 114.8 +/− 61.2, B 101.5 +/− 39.9	549.3 +/− 6.0 lux	CCT not interpreted

**Table 2:** Recognition rate by lighting condition and object position.

Lighting Condition	Position 1	Position 2
Red	0/10 recognized, 0%	0/10 recognized, 0%
Green	10/10 recognized, 100%	10/10 recognized, 100%
Blue	0/10 recognized, 0%	0/10 recognized, 0%
Daylight-like	10/10 recognized, 100%	10/10 recognized, 100%
Halogen	10/10 recognized, 100%	10/10 recognized, 100%

**Table 3:** Frames until object recognition at position 1.

Lighting Condition	*n*	Mean +/− Standard Deviation (SD)	Median	Minimum– Maximum	Recognition
Red	10	1000.0 +/− 0.0	1000.0	1000–1000	0%
Green	10	173.6 +/− 36.0	160.0	126–254	100%
Blue	10	1000.0 +/− 0.0	1000.0	1000–1000	0%
Daylight-like	10	162.0 +/− 37.2	156.0	116–233	100%
Halogen	10	130.6 +/− 22.5	129.5	89–162	100%

**Table 4:** Frames until object recognition at position 2.

Lighting Condition	*n*	Mean +/− Standard Deviation (SD)	Median	Minimum– Maximum	Recognition
Red	10	1000.0 +/− 0.0	1000.0	1000–1000	0%
Green	10	153.5 +/− 27.8	164.0	99–189	100%
Blue	10	1000.0 +/− 0.0	1000.0	1000–1000	0%
Daylight-like	10	196.8 +/− 12.0	194.5	180–217	100%
Halogen	10	181.8 +/− 16.9	187.0	151–200	100%

**Table 5:** Comparison between position 1 and position 2. Positive differences indicate higher frame numbers at position 2.

Lighting Condition	Position 1	Position 2	Difference P2-P1	*p*-Value	Result
Red	1000.0	1000.0	0.0	not calculable	no recognition at either position
Green	173.6	153.5	−20.1	0.225	not significant
Blue	1000.0	1000.0	0.0	not calculable	no recognition at either position
Daylight-like	162.0	196.8	+34.8	0.022	significant
Halogen	130.6	181.8	+51.2	0.001	significant

**Table 6:** Robust Michelson contrast of the ROI at position 1.

Lighting Condition	*n*	Mean +/− SD	Median	Minimum–Maximum
Green	1001	0.8264 +/− 0.0268	0.8242	0.7460–0.8864
Daylight-like	1001	0.8123 +/− 0.0089	0.8144	0.7955–0.8391
Halogen	1001	0.6584 +/− 0.0104	0.6571	0.6322–0.6905
Red	1000	0.4796 +/− 0.0499	0.4545	0.3333–0.5600
Blue	1001	0.3753 +/− 0.0263	0.3636	0.3077–0.4545

## Data Availability

The original contributions presented in the study are included in the article/ Supplementary Materials, further inquiries can be directed to the corresponding authors.

## Supplementary Materials

The following supporting information can be downloaded at: https://www.mdpi.com/article/10.3390/jfb17080384/s1, Raw frame-count data, lighting-condition sensor values and ROI-based contrast parameters used for the analysis.

## Abbreviations

The following abbreviations are used in this manuscript:

**Abbreviation**	**Full term**
AR	Augmented Reality
AI	Artificial Intelligence
ANOVA	Analysis of Variance
CAD/CAM	Computer-Aided Design/Computer-Aided Manufacturing
CCT	Correlated Color Temperature
DC	Direct Current
FDM	Fused Deposition Modeling
fps	Frames per Second
I2C	Inter-Integrated Circuit
ISO	International Organization for Standardization
LED	Light-Emitting Diode
PLA	Polylactic Acid
QR	Quick Response
RGB	Red-Green-Blue
ROI	Region of Interest
SD	Standard Deviation
